# New Mechanisms of Tumor-Associated Macrophages on Promoting Tumor Progression: Recent Research Advances and Potential Targets for Tumor Immunotherapy

**DOI:** 10.1155/2016/9720912

**Published:** 2016-11-16

**Authors:** Qiujun Guo, Zhichao Jin, Yuan Yuan, Rui Liu, Tao Xu, Huamin Wei, Xinyao Xu, Shulin He, Shuntai Chen, Zhan Shi, Wei Hou, Baojin Hua

**Affiliations:** ^1^Department of Oncology, Guang'anmen Hospital, China Academy of Chinese Medicine Sciences, No. 5 Beixiange, Xicheng District, Beijing 100053, China; ^2^Beijing University of Chinese Medicine, No. 11 North Third Ring Road East, Chaoyang District, Beijing 100029, China; ^3^Department of Oncology, Xiyuan Hospital, China Academy of Chinese Medicine Sciences, No. 1 Playground Road, Haidian District, Beijing 100091, China; ^4^Institute of Basic Research in Clinical Medicine (IBRCM), China Academy of Chinese Medicine Sciences, No. 16 Dongzhimen Nanxiaojie, Dongcheng District, Beijing 100700, China

## Abstract

The majority of basic and clinical studies have shown a protumor function of tumor-associated macrophages (TAMs), which represent a large proportion of matrix cells. TAMs promote tumorigenesis, and their number is related to the malignancy degree and poor prognosis of many kinds of tumors. Macrophage plasticity makes it possible to change the tumor microenvironment and remodel antitumor immunity during cancer immunotherapy. Increasing numbers of studies have revealed the effects of TAMs on the tumor microenvironment, for example, via promotion of tumor growth and tumorigenesis and through an increase in the number of cancer stem cells or via facilitation of angiogenesis, lymphangiogenesis, and metastasis. Investigators also proposed tumor-immunological treatments targeting TAMs by inhibiting TAM recruitment and differentiation, by regulating TAM polarization, and by blocking factors and pathways associated with the protumor function of TAMs. This comprehensive review presents recent research on TAMs in relation to prediction of poor outcomes, remodeling of the tumor immune microenvironment, and immunological targeted therapies.

## 1. Introduction

Macrophages are differentiated cells of the mononuclear phagocytic lineage. They are heterogeneous cells with distinct functions and respond differently to various microenvironmental signals and thus have distinct functions. Macrophages—derived from hematopoietic stem cells in bone marrow or from progenitor cells in the embryonal yolk sac—differentiate into two distinct types of macrophages (M*ϕ*), classically activated or M1 M*ϕ* (“killing” phenotype) and alternatively activated or M2 M*ϕ* (“healing” phenotype). Tumor-associated macrophages (TAMs) may represent up to 50% of the tumor mass, and most TAMs have the M2 phenotype due to the signals in the tumor microenvironment, such as IL-4 and TGF-*β* [1, 2]. As generally accepted at present, TAMs play a key role in tumorigenic processes ranging from tumor initiation to acceleration of tumor progression and metastasis.

## 2. TAMs Lead to a Poor Clinical Prognosis and Promote Progression of Various Tumors

### 2.1. TAMs Can Be Considered a Biomarker of Poor Prognosis

TAMs provide a suitable microenvironment for tumor invasion and progression and contribute to the metastasis of tumor cells [3]. Researchers have shown that the existence of TAMs positively correlates with a poor clinical prognosis of various tumors, such as lung cancer, gastric cancer, gynecological tumors, and lymphomas (Table 1). Although there are a few exceptions to this association, such as colorectal carcinoma [4], these data still indicate that TAMs play an essential role in tumorigenesis; accordingly, targeting of TAMs may be a promising method of cancer treatment.

### 2.2. TAMs Reduce the Curative Effect of Chemotherapy

Chemotherapy is a conventional treatment modality for cancer patients. Although chemotherapies have strong effects on some kinds of tumors, such as small cell lung cancer [5] and lymphomas [6], the overall response rate is less than satisfactory for many tumors [7]. Furthermore, drug resistance, tumor recurrence, and metastasis make tumor treatment more difficult.

TAMs were found to help reduce the effects of chemotherapy. Infiltration by CD68^+^ and CD163^+^ TAMs is associated with a poor response to chemotherapy in patients with esophageal cancer [8, 9]. TAMs are also recruited to the pancreatic tumor site and stimulate adenocarcinoma cells to express high levels of cytidine deaminase, which catabolizes the bioactive form of gemcitabine and reduces the sensitivity of cancer cells to chemotherapy [10]. Yang et al. [11] also found that TAMs can induce chemotherapeutic drug resistance of a breast tumor through the IL-10/STAT3/Bcl-2 signaling pathway, and another study showed that TAMs correlate with the resistance to tamoxifen, an endocrine therapy drug for postmenopausal breast cancer patients [12].

Epidermal growth factor receptor tyrosine kinase inhibitors (EGFR-TKIs) are novel treatments of lung cancer with EGFR mutation and have higher specificity and fewer side effects than traditional treatments; M2-polarized TAMs also correlate with the decreased responsiveness to EGFR-TKI treatment in patients with advanced lung adenocarcinoma [13].

### 2.3. TAMs Cause an Unfavorable Outcome of Radiotherapy

Radiotherapy (RT) is a localized therapy that is highly effective at killing primary tumor cells located within the field of the radiation beam. Despite sophisticated techniques for radiation delivery as well as the combination of radiation with chemotherapy, tumors can recur [14].

TAMs are abundantly recruited to tumors after irradiation and may modulate cancer cells' responses to therapy [15]. It has been suggested that macrophages may be involved in modulating the late effects of radiotherapy [16]. Various studies indicate a correlation between high TAM numbers and poor tumor responses to irradiation in mouse models [17]. Furthermore, Shiao et al. [18] reported that regrowth of mammary tumors after radiotherapy correlates with the influx of Th2-polarized macrophages. After tumor irradiation, DNA damage, cell death, and increased tumor hypoxia may promote production of VEGF, SDF-1, and CSF-1, resulting in the recruitment, infiltration, and retention of monocytes/macrophages within the tumor [14]. The recruited heterogeneous populations of TAMs release proangiogenic cytokines and metalloproteinases to promote blood vessel formation within the tumor [19]. Teresa Pinto et al. [20] also found that irradiated macrophages promote cancer cell invasiveness and cancer cell-induced angiogenesis. Besides, the abscopal effect may be a crucial factor in evaluation of the prognosis associated with radiotherapy, and TAMs can release secondary bystander signals and play a key role in the secondary bystander effect of photon irradiation [21]. Targeting TAMs or TAM-associated signaling to enhance the potency of radiotherapy has been similarly demonstrated in several other studies [14, 22–24]. Inhibition of TAM recruitment after radiotherapy by neutralizing CSF-1 or blocking CSF-1R kinase activity may significantly slow tumor regrowth [15].

## 3. TAMs Remodel the Tumor Microenvironment

### 3.1. TAMs Ensure an Immunosuppressive Tumor Microenvironment

The tumor immune microenvironment is mainly formed by such immune cells as macrophages, T lymphocytes, natural killer cells (NK cells), dendritic cells, neutrophils, and myeloid-derived suppressor cells (MDSCs) [47, 48]. As a kind of immunosuppressive cell subgroup, TAMs express chemokines and cytokines and contribute to the immunosuppressive tumor microenvironment (ITM) [49]. Chemokines (such as CCL5, CCL22, and CCL20) secreted by TAMs recruit regulatory T (Treg) cells, whereas cytokines (such as IL-10 and TGF-*β*) induce Treg cells. Besides, TAMs may inhibit the antitumor effect of tumor-infiltrating T cells and NK cells [50, 51] and promote ITM synergistically with MDSCs, tumor-associated dendritic cells, and neutrophils [47, 52–54]. TAMs may suppress T-cell function by secreting specific enzymes such as nitric-oxide synthase (NOS) and arginase (ARGI) [55, 56]. In addition, TAMs express ligands for receptors called PD-1 and CTLA-4 (such ligands as PD-L1 and B7-H1), which after activation suppress cytotoxic functions of T cells, NKT cells, and NK cells [57].

### 3.2. TAMs Promote Tumorigenesis

Cancer can be considered a nonresolving inflammatory disease. Up to 20% of all cancers arise in association with chronic inflammation, and almost all solid tumors contain inflammatory infiltrates [58]. TAMs, as the major immune cells in a tumor, have a broad impact on tumor initiation. Macrophages can play contrasting roles in cancer depending on their phenotype. M1-type macrophages have the potential to contribute to the earliest stages of neoplasia, whereas M2-type macrophages usually get involved after a tumor progresses and grows [59]. DNA damage is the major mechanism of tumorigenesis induced by inflammation. The free radicals produced by TAMs can lead to DNA damage, causing mutations that predispose the affected individual to cancer. An example of this macrophage-mediated induction of tumorigenesis is that Crohn's disease dramatically increases the risk of colorectal cancer [60–63]. Besides, chronic infection with viruses such as hepatitis B virus in the liver or with bacteria like* Helicobacter pylori* in the stomach or continuous exposure to irritants such as asbestos in lungs is casually associated with cancer initiation.

### 3.3. TAMs Facilitate Metastasis

Metastasis is responsible for more than 90% of cancer mortality yet remains the least understood stage of tumor progression. TAMs have been shown to be key players in metastasis and mainly participate in several steps including epithelial-to-mesenchymal transition (EMT), local invasion and intravasation into the vasculature, transit through the circulatory system, extravasation and seeding in the premetastatic niche, and finally survival and growth at the metastatic site [64, 65]. TAMs can promote progression and metastasis through the release of a variety of chemokines, inflammatory factors, and growth factors. It is reported that CD68^+^HLA-DR^+^ TAMs in hepatocellular carcinoma (HCC) can promote migration of HCC cells via the NF-*κ*B/FAK pathway [66]. Specifically, TAM-derived IL-6 and IL-8 enhance invasive activity of LoVo cells induced by PRL-3 in a KCNN4 channel-dependent manner [67].

In breast tumors, TAMs are recruited to pulmonary metastases by CCL2 and enhance extravasation, seeding, and persistent growth of tumor cells in part via expression of VEGF [68]. CCL2 synthesized by a tumor and stroma also triggers a prometastatic chemokine cascade (involving CCL3 signaling via CCR1) that is required for efficient metastasis [69]. Besides, TAMs binding to VCAM-1 expressed on breast cancer cells can promote tumor cell survival in lungs [70]. The bidirectional cross-talk between cancer cells and TAMs constitutes a microanatomic landmark, which is defined as the tumor microenvironment of metastasis (TMEM), whereas TMEM density is positively associated with the risk of distant organ metastases [71].

EMT is a process via which epithelial tumor cells lose epithelial features and gain mesenchymal phenotypes [72]. EMT is considered the key step via which tumor cells gain the greater capacity for invasiveness and metastasis. TAMs can strongly express many kinds of cytokines that can induce EMT, such as TGF-*β* and IL-6 [73]. TNF-*α* secreted by TAMs has been shown to activate NF-*κ*B-mediated transcription of Snail1 and Zeb, which leads to diminished E-cadherin expression on tumor cells [74]. One report suggests that M2 macrophages may induce EMT by regulating TLR4/IL-10 signaling in pancreatic cancer cells [75]. TAMs may also secrete EGF-like ligands/factors that activate the EGFR pathway in cancer cells, thus promoting EMT, and this macrophage-induced EMT is significantly inhibited by treatment of A549 cancer cells with JWH-015 [76].

CCL18 released from TAMs can induce EndMT in endothelial cells to produce a different differentiated phenotype, which may lead to a loss of cell-cell junctions as well as enhanced invasiveness and migratory capacity [77]. These data confirm a strong cross-talk between macrophages and tumor progression, mainly through stimulation of EMT.

### 3.4. TAMs Promote Angiogenesis and Lymphangiogenesis

The growth and spread of neoplasms depend on angiogenesis and lymphangiogenesis in the tumor microenvironment. With neoplastic progression, increasing numbers of blood and lymphatic vessels provide supply channels for tumor tissues. On the other hand, it is reported that these mounting vessels provide a route for the lymph-nodal and distant metastases of tumor cells [78–81]. The infiltration by TAMs is associated with extensive angiogenesis, which contributes to a poor prognosis in primary cancers. Multiple factors are involved in angiogenesis: hypoxia, hyperosmosis, and proangiogenic factors such as vascular endothelial growth factor (VEGF), transforming growth factor beta (TGF-*β*), cyclooxygenase 2 (COX-2), platelet-derived growth factor (PDGF), epidermal growth factor (EGF), angiopoietins (Ang), and chemokines [82–86]. CCL18 released from TAMs can promote angiogenesis and tumor progression in breast cancer [70]. TAMs can also synthesize Wnt7b, which targets vascular endothelial cells by stimulating their production of VEGF resulting in the angiogenic switch [87]. One of the major angiogenesis-inducing factors, pro-matrix metalloproteinase-9 (proMMP-9), is supplied to the tumor microenvironment by TAMs [88]. It has been demonstrated that matrix metalloproteinase 9 (MMP-9) plays a crucial role in tumor angiogenesis and metastasis by turning on the angiogenic switch in avascular tumors and by mediating the development and maintenance of distinct neovascular networks sustaining tumor cell intravasation [89, 90]. Hypoxia causes a tumor microenvironment overexpressing hypoxia-inducible factor 1*α* (HIF-1*α*), the primary transcription factor involved in homeostasis of oxygen concentration. HIF-1*α* can activate transcription of genes encoding angiogenic growth factors and stimulating endothelial cells (ECs), thus leading to angiogenesis [75]. TAMs' localization to hypoxic tumor areas is controlled by the Sema3A/neuropilin-1 signaling axis, leading to plexinA1/plexinA4-dependent VEGFR1 activation and promotion of tumor growth and metastasis [91]. In addition, TAMs can increase the expression of HIF-1*α* and promote endothelial-tube formation in colon cancer [92]. Besides, Laoui et al. found that hypoxia specifically lowers hypoxia-sensitive gene expression and angiogenic activity in the MHC-II^lo^ TAM subset instead of altering differentiation of the TAM population [93].

Coordination of the lymphatic microvascular network with the blood microvasculature is involved in normal physiological functions, such as local tissue fluid balance, tissue perfusion, and immune surveillance [94–96], and gives more weight to lymphangiogenesis during tumor growth and cancer metastasis [72]. A number of studies have revealed lymphatic microvessel density (LMVD) to be an independent prognostic factor for solid tumors [97–100]. Additionally, TAMs are reportedly involved in lymphangiogenesis in malignant tumors [101]. Zhang et al. found that TAMs in lung adenocarcinoma are associated with poor prognoses resulting from accelerated lymphangiogenesis and lymph node metastasis [102].

Tumor cells induce formation of lymphatic vessels via the lymphatic system metastasis through VEGF-C, VEGF-D, and other cytokines [103]. The expression of VEGF-C and VEGF-D by TAMs suggests that TAMs are intimately involved in the generation of tumor lymphatic vessels [104]. M2-polarized TAM infiltration of RLNs is significantly associated with nodal lymphangiogenesis, and node-infiltrating M2-polarized TAMs may facilitate nodal lymphangiogenesis via production of VEGF-C [105].

### 3.5. TAMs and Cancer Stem Cells (CSCs)

CSCs or cancer-initiating cells are defined as a small subpopulation of cancer cells with the capacity for self-renewal and pluripotency. CSCs are necessary for initiation of new tumor growth at distant sites. Currently, many studies support the notion that CSCs, which have many features of stem cells, are responsible for the poor prognosis of patients by promoting tumor recurrence and metastasis [106, 107].

TAMs can regulate the plasticity of CSC phenotypes and functions. Recently, some of TAMs-CSCs interrelations were confirmed experimentally. TAMs were found to release milk-fat globule EGF-VIII, which activates the CSC-specific pathways—STAT3, Hedgehog, and Sonic—and strongly amplifies drug resistance and tumorigenicity of CSCs [108]. MFG-E8 and IL-6 from TAMs can also synergistically mediate tumorigenicity and drug resistance in subsets of CSCs including those in primary human tumors [109]. In experiments with murine mammary CSCs, Yang et al. reported that drug resistance of CSCs is associated with new EGFR/STAT3/Sox-2 paracrine signaling pathway activity that is realized via a complex interplay between CSCs and TAMs [110]. It is reported that TAMs have effects on gastric CSCs in omental milky spots and on lymph node micrometastasis, which is mainly mediated by activation of MCP-1, COX-2, PGE-2, IL-10, IFN-*γ*, and VEGF and by downregulation of IL-4, TGF-*β*, MMP-2, and MMP-9 [111]. It has been suggested TAMs may promote CSC-like properties via TGF-*β*1-induced EMT and may advance the research on the prognosis of HCC [112]. Furthermore, microglia and brain TAMs serve as mediators of glioma stem-like cell (GSLC) properties, producing high levels of TGF-*β*, which makes GSLCs more invasive [113]. Direct cell-cell interactions of TAMs with CSCs via Thy1 and Eph4A receptors have been reported to induce activation of NF-*κ*B, which in turn sustains the CSCs state [114].

**Figure 1:**
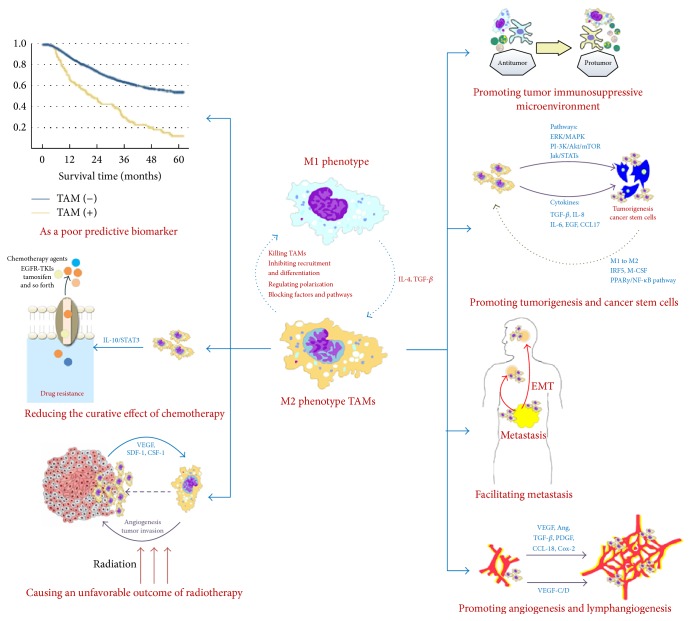
TAMs promote tumor progression.

The functional and phenotypic heterogeneity of CSCs themselves in turn affects the pathophysiological activities of TAMs. It seems that active CSCs should be able to promote the M1-to-M2 conversion, induce formation of new vasculature via VEGF release, and build CSC-protective niches via tissue-repair pathways [2]. OCSCs may promote the M2 polarization of macrophages through the PPAR*γ*/NF-*κ*B pathway [115]. Chemoresistant CSCs may promote M2 macrophage differentiation through interferon-regulatory factor-5- (IRF5-) and macrophage-colony stimulating factor- (M-CSF-) dependent mechanisms [116]. Chen et al. reported that embryonic stem cells can promote macrophage survival and M2-like activation, which are critically important for teratoma angiogenesis and development [117] (Figure 1).

TAMs can be considered a biomarker of poor prognosis and reduce the curative effect of chemotherapy and radiotherapy. In terms of mechanisms, TAMs may promote a tumor immunosuppressive microenvironment, tumorigenesis, angiogenesis, and lymphangiogenesis and can facilitate metastasis.

## 4. Treatments Targeting TAMs

Immunotherapy acts in a fundamentally different way in comparison with classical therapies. Rather than destroying tumor cells directly, immunotherapy promotes tumor cell killing via the immune response of the host. This result can be achieved directly via the main effectors of the immune system, such as macrophages. Evidence reviewed by Mills et al. indicates that modulation of macrophage responses is a breakthrough that will facilitate successful immunotherapy [118]. In a sorafenib-resistant tumor model, photoimmunotherapy targeting TAMs was found to inhibit the tumor growth and metastasis [119]. Therefore, TAMs have become promising therapeutic targets for cancer treatment. In recent years, many researchers focused on cancer immunotherapy related to TAMs (Table 2).

### 4.1. Killing of TAMs or Inhibition of TAM Recruitment and Differentiation

Strategies to deplete TAMs have been successful in experimental settings and are now considered a promising therapeutic approach in the clinic [120]. The current approach to targeting of TAMs that has shown efficacy is inhibition of CSF-1/CSF-1R signaling because this axis is required for macrophage survival [121, 122]. For instance, targeting of CSF-1/CSF-1R alters macrophage polarization and blocks glioma progression [123]. RG7155, a monoclonal antibody that inhibits CSF-1 receptor (CSF-1R), can provide significant clinical benefits and offer a therapeutic option other than surgical treatment to patients with a diffuse-type giant cell tumor (Dt-GCT) [124].

An RNA aptamer that blocks the murine or human IL-4 receptor-*α* (IL4R*α* or CD124) can preferentially target TAMs and unexpectedly promote their elimination, an effect that is associated with an increased number of tumor-infiltrating T cells and a reduction in tumor growth [125]. Allavena et al. have recently demonstrated that trabectedin, a licensed and commercially available anticancer agent, is selectively toxic to TAMs because of activation of caspase 8-dependent apoptosis [126, 127]. Membrane-permeating drugs can also induce apoptosis in macrophages; this effect may be exploited for the depletion of TAMs [128]. Inhibiting recruitment of macrophages to neoplastic lesions is one of the therapies targeting TAMs. The antitumor agent dequalinium-14 in addition to its antitumor effect can reduce macrophage motility, inhibit macrophage infiltration of irradiated tumors, and reduce the extent of metastasis in locally irradiated mice [14]. Besides, TAMs derive from monocytes and have the capacity for differentiation; therefore, a combination therapy blocking differentiation may be required for effective targeting of these cells [129]. Interferon *γ* may induce recruitment of monocytes/macrophages into the tumor microenvironment but inhibits their differentiation into TAMs* in vivo*; this effect may reduce the concentration of VEGF and angiogenesis in a tumor [130].

### 4.2. Regulation of TAM Polarization

TAMs are typically designated as “alternatively activated” noninflammatory M2-type macrophages, in contrast to the “classically activated” inflammatory M1 type. TAMs coexist with tumors and function as an accomplice in the promotion of tumor progression, especially after being programmed and polarized into a proangiogenic/immunosuppressive (M2-like) phenotype by the tumor microenvironment [1, 75]. Unlike Th1 and Th2 cells, M1 and M2 macrophages are not stably differentiated subsets and can switch the phenotype [131]. In this case, TAMs represent an ideal therapeutic target for blocking tumor progression after being reprogrammed and repolarized to express a proimmunity (M1-like) phenotype [132]. Dimethyl sulfoxide can suppress mouse 4T1 breast cancer growth by modulating TAM differentiation [133]. It was found that PA-MSHA can reeducate CD163^+^ TAMs into M1 macrophages through the TLR4-mediated pathway in MPE [27]. M-CSFR signaling was found to govern the phenotype of M2-like MHC-II^lo^ TAMs, and its blockade results in preferential differentiation of monocytes into M1-like MHC-II^hi^ TAMs [134]. Interferon *γ* and celecoxib can inhibit lung tumor growth by modulating the M2/M1 macrophage ratio in the tumor microenvironment [135].

### 4.3. Blocking of Factors and Pathways Associated with the Protumor Function of TAMs

The ability of TAMs to accelerate vessel growth is mediated by increased secretion of several proangiogenic factors. Therapeutic success in blocking these protumor activities in preclinical models and early clinical trials highlighted macrophages as effective targets of combination cancer therapy [136]. It is reported that somatostatin derivate (smsDX) can attenuate the TAM-stimulated proliferation, migration, and invasiveness of prostate cancer via NF-*κ*B regulation [137]. Luteolin can also inhibit recruitment of monocytes and migration of Lewis lung carcinoma cells by suppressing chemokine (C-C motif) ligand 2 expression in TAMs [138]. Besides, it has been shown that a cannabinoid receptor 2 agonist inhibits macrophage-induced EMT in non-small cell lung cancer via downregulation of the EGFR pathway [69].

## 5. Conclusions

TAMs comprise an important part of the tumor microenvironment, and their mobilization into the tumor microenvironment plays a key role in malignant progression. Studies have shown that TAMs lead to a poor clinical prognosis and promote progression of various tumors; these cells also correlate with an unfavorable outcome following therapy. After accumulation in tumor tissues, TAMs can remodel the tumor microenvironment to promote matrix remodeling and promote tumor growth and increase angiogenesis CSC-associated tumor progression. With rapid progress in the understanding of TAM functions, new therapeutic approaches against tumors have been developed, such as inhibition of TAM recruitment or suppression of TAM survival, regulation of TAM polarization, reprogramming of TAMs into the antitumor M1 phenotype, and blocking of factors and pathways associated with the protumor function of TAMs. Thus, the increasing knowledge about the biological effects of TAMs and the tumor microenvironment may lead to novel cancer therapies.

## Figures and Tables

**Table 1:** TAMs associated with poor prognosis.

Cancer	IHC marker	Prognostic outcome	Ref.
Hormone receptor-positive breast cancer	Intratumoral TAMs	Poor DFS	[25]
Triple-negative breast cancer	CD68^+^ TAMs	Poor OS and DFS	[26]
Gastric cancer	CD204^+^ TAMs combined with Osteopontin (OPN)	Poor 5-year OS	[27]
Lung cancer induced malignant pleural effusion (MPE)	CD163^+^ TAMs in MPE	Poor progression-free survival	[28]
Non-small cell lung cancer	TAMs combined with OPN	Lower DFS and OS	[29]
	TAMs combined with IL-6, CSF-1	Lower 5-year survival rate	[9]
Esophageal cancer undergoing neoadjuvant chemotherapy	CD68^+^ and CD163^+^ TAMs	Tumor depth, lymphatic invasion, venous invasion, and poor response to chemotherapy	[6]
Oral squamous cell carcinoma	CD68^+^ TAMs or combined with ALDH1, CD44, SOX2, IL-10	High tumor grade, lymph node metastasis, shorter OS and DFS, and poor clinical stage	[30–32]
Nonfunctional pancreatic neuroendocrine tumors (NF-PNETs)	CD68^+^ TAMs combined with Ki-67 index	High risk of recurrence	[33]
Glioma	CD206^+^ TAMs	Lower PFS and OS	[34]
Colorectal cancer	CD40^+^ TAMs or combined with urokinase-type plasminogen activator receptor	Lower OS, lymph node, and distant metastasis	[35, 36]
Pancreatic ductal adenocarcinoma	CD204^+^ TAMs combined with CD44/CD133	Lower PFS and OS	[37]
Advanced epithelial ovarian cancer	CD68^+^ and CD163^+^ TAMs	Poor PFS and OS	[38]
Bladder carcinoma	CD68^+^ TAMs	Poor recurrence-free survival	[39]
Triple-negative endometrial endometrioid adenocarcinoma	CD68^+^ TAMs combined with overexpression of EGFR	Poor OS	[40]
Prostate cancer	CD68^+^ TAMs	Poorly differentiated disease but no association with biochemical recurrence after radical prostatectomy	[41]
		Poor OS	[42]
Classical Hodgkin lymphoma	CD68^+^ and CD163^+^ TAMs	High risk of treatment failure with ABVD chemotherapy	[43]
Primary central nervous system lymphoma (PCNSL)	CD68^+^ and CD163^+^ TAMs	Inferior PFS but no association with OS	[44]
Peripheral T-cell lymphoma	CD68^+^ TAMs combined with VEGF	Poor OS	[45]
Diffuse large B-cell lymphoma	CD68^+^ TAMs	Poor treatment outcome and poor median survival time	[46]

PFS: progression-free survival, DFS: disease-free survival, and OS: overall survival.

**Table 2:** Immunotherapies targeting TAMs.

Therapeutic approaches	Cancer	Drugs	Ref.
*Killing TAMs or inhibiting TAM recruitment and differentiation*			
Blocking the murine or human IL-4 receptor *α* (IL4R*α* or CD124)	—	RNA aptamer	[125]
Inducing apoptosis in TAMs via Kv1.3 and Kv1.5 potassium channels	—	Membrane-permeant drugs	[118]
Selectively cytotoxic for TAMs and their circulating precursors (monocytes) by activating caspase 8-dependent apoptosis	Sarcoma and ovarian carcinoma	Trabectedin	[126]
Reducing macrophage motility, inhibiting macrophage infiltration of irradiated tumors	Colon carcinoma	Dequalinium-14	[12]
Suppressing of tumor-associated macrophage differentiation	Gallbladder cancer	Interferon-*γ*	[120]
*Regulation of TAM polarization*			
Reversing TAM orientation and polarization from M2- to M1-type TAMs	Breast cancer	Dimethyl sulfoxide	[124]
Reeducating CD163^+^ TAMs to M1 macrophages through TLR4-mediated pathway	MPE	PA-MSHA	[23]
Shifting the M1/M2 TAMs balance by M-CSFR signaling blockade	Lung carcinoma and breast carcinoma	Anti-M-CSFR antibody	[125]
Modulating the M2/M1 macrophage ratio	Lung cancer	IFN*γ* and/or celecoxib	[126]
*Blocking factors and pathways associated with protumor function of TAMs*			
Inhibiting the paracrine loop between TAM and PCa cells via NF-*κ*B regulation	Prostate cancer	Somatostatin derivate (smsDX)	[128]
Suppressing chemokine (C-C motif) ligand 2 expression in tumor-associated macrophage	Lung carcinoma	Luteolin	[129]
Inhibiting macrophage-induced EMT by downregulation of EGFR pathway	Non-small cell lung cancer	Cannabinoid receptor-2 agonist	[69]
