# Corrigendum to “New Mechanisms of Tumor-Associated Macrophages on Promoting Tumor Progression: Recent Research Advances and Potential Targets for Tumor Immunotherapy”

**DOI:** 10.1155/2018/6728474

**Published:** 2018-07-29

**Authors:** Qiujun Guo, Zhichao Jin, Yuan Yuan, Rui Liu, Tao Xu, Huamin Wei, Xinyao Xu, Shulin He, Shuntai Chen, Zhan Shi, Wei Hou, Baojin Hua

**Affiliations:** ^1^Beijing University of Chinese Medicine, No. 11 North Third Ring Road East, Chaoyang District, Beijing 100029, China; ^2^Department of Oncology, Guang'anmen Hospital, China Academy of Chinese Medicine Sciences, No. 5 Beixiange, Xicheng District, Beijing 100053, China; ^3^Department of Oncology, Xiyuan Hospital, China Academy of Chinese Medicine Sciences, No. 1 Playground Road, Haidian District, Beijing 100091, China; ^4^Institute of Basic Research in Clinical Medicine (IBRCM), China Academy of Chinese Medicine Sciences, No. 16 Dongzhimen Nanxiaojie, Dongcheng District, Beijing 100700, China

In the article titled “New Mechanisms of Tumor-Associated Macrophages on Promoting Tumor Progression: Recent Research Advances and Potential Targets for Tumor Immunotherapy” [1], the order of the first two affiliations was reversed. The revised authors' list and affiliations are shown above.
